# A comparison of National Healthcare Safety Network surveillance definitions to physician review for central-line–associated bloodstream infections

**DOI:** 10.1017/ice.2023.128

**Published:** 2023-12

**Authors:** Justin J. Kim, HeeEun Kang, Asif N. Khan, Rebecca Wang, Stephanie C. Casale, Caitlin M. Adams Barker, Michael S. Calderwood, Kathleen O. Stewart

**Affiliations:** 1 Section of Infectious Disease and International Health, Dartmouth Hitchcock Medical Center, Lebanon, New Hampshire; 2 Collaborative Healthcare-associated Infection Prevention Program, Dartmouth Hitchcock Medical Center, Lebanon, New Hampshire; 3 Geisel School of Medicine at Dartmouth College, Hanover, New Hampshire; 4 The Dartmouth Institute for Health Policy and Clinical Practice, Dartmouth Hitchcock Medical Center, Lebanon, New Hampshire; 5 Quality Assurance and Safety, Dartmouth Hitchcock Medical Center, Lebanon, New Hampshire

## Abstract

For 147 hospital-onset bloodstream infections, we assessed the sensitivity, specificity, positive predictive value, and negative predictive value of the National Healthcare Safety Network surveillance definitions of central-line–associated bloodstream infections against the gold standard of physician review, examining the drivers of discrepancies and related implications for reporting and infection prevention.

A central-line–associated bloodstream infection (CLABSI) is a healthcare-associated infection resulting in increased morbidity, mortality, and healthcare costs. CLABSIs are strictly defined by surveillance rules of the Centers for Disease Control and Prevention (CDC) National Healthcare Safety Network (NHSN).^1^ Although the NHSN surveillance definitions have been internally validated in multiple studies,^2^ few studies have assessed their external validity.^3^ We assessed the sensitivity, specificity, positive predictive value (PPV), and negative predictive value (NPV) of the NHSN definitions against a gold standard of physician review of hospital-onset bloodstream infections (BSIs) with eligible central lines at our institution. We have described the drivers of any discrepancies between the surveillance and clinical designations as well as the implications of these discrepancies on the reporting of quality metrics and opportunities for infection prevention.

## Material and methods

In total, 147 hospital-onset BSIs (ie, bacteremia occurring on hospital day 3 or later) with eligible central lines (ie, central line present for >2 calendar days after first being accessed, until the calendar day following line removal or patient discharge, whichever sooner) occurred at our 400-bed, rural, tertiary-care, academic medical center between July 2019 and June 2022. As part of routine hospital operations, all BSIs were investigated by at least 2 infection preventionists using NHSN surveillance definitions and were given a designation of CLABSI or bacteremia secondary to a primary infection.^1^ Additional input was solicited from other infection preventionists on the team, the medical director of infection prevention, and via emails to the NHSN for difficult cases.

For comparison, physicians independently reviewed notes, laboratory studies, and imaging from the medical record to designate BSIs as CLABSI or secondary to a primary infection. Infectious disease fellows conducted a first review (H.K. and A.N.K.), and an infectious disease attending physician conducted a second review (J.J.K.) of all BSIs. Another infectious disease attending physician conducted a third review (R.W.) in cases of disagreement. We assessed interrater reliability between the first 2 reviews using the Cohen κ.

We calculated the sensitivity, specificity, PPV, and NPV of the NHSN definitions for CLABSIs compared to the gold standard of physician review. We examined discrepancies between the NHSN definitions and physician review. This study was designated “not human research” by the Dartmouth-Hitchcock Institutional Review Board.

## Results

**Figure 1.**
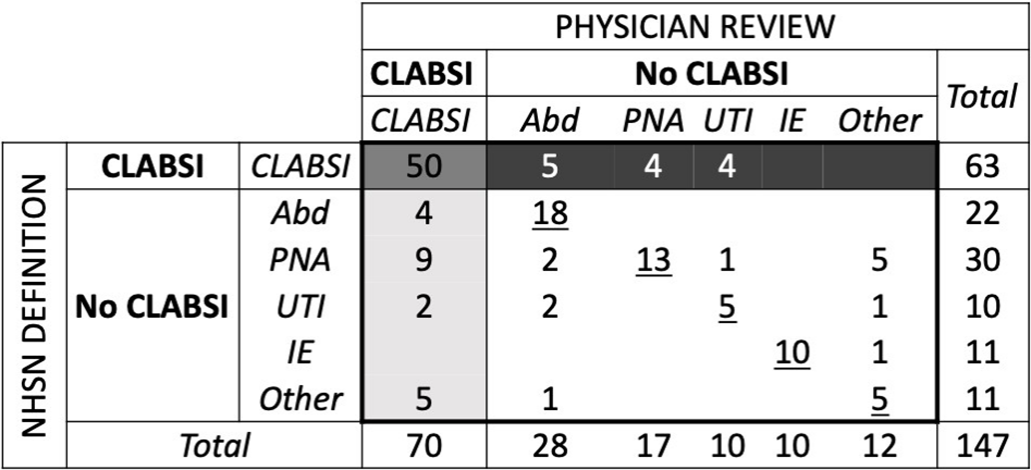
Confusion matrix comparing NHSN CLABSI surveillance definitions to physician review. The bolded center box draws attention to a 2×2 table where true positives are highlighted with medium-gray shading. True negatives are white with the underlined diagonal demonstrating agreement on secondary infection source. False positives are dark gray and false negatives are light gray. Blank cells have a count of zero. Note. CLABSI, central-line–associated bloodstream infection; NHSN, National Healthcare Safety Network; Abd, abdominal infection; PNA, pneumonia; UTI, urinary tract infection; IE, infective endocarditis; other, skin and soft tissue, surgical site, bone and joint, central nervous system, reproductive system, unclear source, and contaminants.

A confusion matrix summarizing our findings is provided in Figure 1. By physician review, 70 (48%) of 147 BSIs were CLABSIs, whereas 28 (19%) were secondary to an abdominal infection, 17 (12%) were pneumonia, 10 (7%) were urinary tract infection (UTI), and 10 (7%) were infective endocarditis. The remaining 12 BSIs were secondary to other sources (ie, 4 were secondary to skin and soft-tissue or surgical site infections and 2 were secondary to bone and joint infections), had an unclear source (n = 5), or contaminant (n = 1). By NHSN surveillance definitions, 63 (43%) of 147 BSIs were CLABSIs, whereas 22 (15%) were secondary to an abdominal infection, 30 (20%) were secondary to pneumonia, 10 (7%) were secondary to UTI, and 11 (7%) were secondary to infective endocarditis. The remaining 11 BSIs were secondary to other sources: 4 bone and joint infections, 4 skin and soft-tissue or surgical-site infections, 2 central nervous system infections, and 1 reproductive system infection. Compared against physician review, the NHSN definitions of CLABSI had sensitivity of 71%, specificity of 79%, PPV of 97%, and NPV of 76%. The NHSN definitions misclassified 20 (14%) of 147 BSIs as being secondary to a primary infection instead of a CLABSI (ie, false negatives), whereas the NHSN definitions misclassified 13 (9%) of 147 BSIs as being a CLABSIs instead of secondary to a primary infection (ie, false positives). The reasons for these discrepancies are summarized in Table 1.

**Table 1.** Discrepancies Between NHSN CLABSI Surveillance Definitions and Physician Review

Discrepancy		Potential Explanation
Physician Review	NHSN Definition	
CLABSI (n=20)	BSI secondary to abdominal infection (n=4)	• By physician review, organisms (ie, 2 *Enterococcus faecium*, 1 *Staphylococcus aureus*, and 1 *Stenotrophomonas maltophilia*) more consistent with a line infection (n=4)
	BSI secondary to pneumonia (n=9)	• BSI (ie, 3 *S. aureus*, 1 *Klebsiella pneumoniae*, 1 *Fusobacterium* spp, 1 *Burkholderia* spp) met NHSN pneumonia definition with supportive imaging without a matching respiratory culture (n=6) • BSI (ie, 2 *S. aureus*, 1 *Candida albicans*) met NHSN pneumonia definition with a matching respiratory culture, but felt to be line-related from physician review (n=3)
	BSI secondary to UTI (n=2)	• *S. aureus* in urine and blood more compatible with line or skin infection (n=1) • *Enterococcus faecalis* in urine and blood more compatible with line infection (n=1)
	BSI secondary to other source (n=5)	• Microbiology (ie, *E. faecium, Escherichia coli,* and *Acinetobacter baumanii*) not felt to be the cause of cellulitis (n=1) • *S. aureus* bacteremia thought to be a distinct event following a bone and joint infection (n=2) • Coagulase negative staphylococci thought to have seeded the shoulder (n=1) • *S. aureus* thought to have seeded the central nervous system (n=1)
BSI secondary to abdominal infection (n=5)	CLABSI (n=13)	• Clinical evidence for BSI (ie, 1 *E. coli,* 1 *Klebsiella oxytoca* and *Enterobacter cloacae,* and 1 *K. pneumoniae*) from translocation did not meet threshold for NHSN definition of gastrointestinal tract or intra-abdominal infection (n=3) o Bowel obstruction (n=1) o Common bile duct dilation, (n=1) o Gastrointestinal bleed (n=1) • Fluid collection on imaging (ie, pancreatic fluid collections) did not meet threshold for NHSN definition of intra-abdominal infection causing BSI (1 *K. pneumoniae*, 1 *C. albicans*) (n=2)
BSI secondary to pneumonia (n=4)		• Threshold value for respiratory cultures (ie, 3 *S. aureus*, 1 *Serratia marcescens*) did not meet NHSN definition of pneumonia (eg, rare or few) (n=4)
BSI secondary to UTI (n=4)		• Urine culture (ie, 1 *E. coli*, 1 *K. pneumoniae*, 2 *Pseudomonas aeruginosa*) could not be used to support a designation of urinary system infection (eg, pyelonephritis) by NHSN (n=4)

Note. NHSN, National Healthcare Safety Network; CLABSI, central-line–associated bloodstream infection; BSI, bloodstream infection; UTI, urinary tract infection.

Interrater reliability was high between the first and second physician reviews, with agreement on 138 (94%) designations (κ = 0.91). The first and second physician reviews disagreed most frequently over whether a BSI was a CLABSI (n = 6) versus secondary to a primary infection (ie, 2 abdominal infections, 2 pneumonia cases, and 2 UTIs).

## Discussion

Our findings suggest that the NHSN surveillance definitions for CLABSI have moderately high sensitivity, specificity, PPV, and NPV compared to the gold standard of physician review, in part because the degree of underreporting (ie, 14% false negatives) was offset by a comparable degree of overreporting (ie, 9% false positives). Underreported cases are not as closely scrutinized in the daily work of infection prevention and represent potentially missed opportunities for the prevention of CLABSIs. Thus, a new quality metric is currently being considered to include all hospital acquired bacteremia, regardless of the presence of a central line.^4^ A major driver of underreporting was the misclassification of BSIs as being secondary to pneumonia, particularly in cases in which pulmonary imaging was the only evidence supporting a diagnosis of pneumonia, accounting for 6 (30%) of 20 of false negatives. However, there are other reasons for chest imaging to be abnormal in hospitalized patients (eg, acute heart failure), and a matching organism in a respiratory specimen could support the attribution of a BSI to pneumonia in the correct clinical context.^5^ Additionally, 10 (50%) of 20 false negatives had *Staphylococcus aureus* bacteremia, suggesting that clinicians typically attribute *S*. *aureus* (ie, a skin commensal that frequently colonizes catheters) to CLABSI, whereas the NHSN definition may misclassify these BSIs as being secondary to other infections.^6^

Even though overreported cases receive similar attention as appropriately reported cases, overreporting is a dissatisfier for frontline healthcare providers whose buy-in is essential for the implementation of key infection prevention practices. The major drivers of overreporting were the misclassification of abdominal infections, pneumonia, and UTIs as NHSN CLABSIs. In clinical practice, BSIs are commonly attributed to translocation in the setting of distension from obstruction (eg, of the colon or common bile duct) or mucosal injury from inflammation (eg, from a gastrointestinal bleed or colitis) in immunocompetent hosts. However, mucosal barrier injury can only be invoked for immunocompromised patients with neutropenia or graft-versus-host disease in the current NHSN definitions.^7^ Although stool and tissue cultures are not often obtained in clinical practice, select imaging studies could be used as objective evidence for BSIs from mucosal barrier injury organisms, as proposed in another study.^3^ For pneumonia, BSIs are commonly attributed to a respiratory source using lower thresholds of growth of a matching organism from respiratory cultures (eg, <10^3^ colony-forming units per milliliter, few, or 1+), particularly in critically ill patients who may be receiving empiric antibiotics before bronchoscopy.^5^ For UTIs, urinary cultures are frequently used to support a diagnosis of pyelonephritis without imaging.^8,9^ Moreover, pyelonephritis is not infrequently accompanied by bacteremia, and blood and urine cultures are rarely discordant.^10^ Even in the absence of typical symptoms, a positive urine culture might warrant treatment in the presence of neutropenia, chronic obstruction from malignancy, or undifferentiated critical illness.

To our knowledge, this study is one of the few external validation studies of the NHSN surveillance definitions for CLABSI.^3^ The major strength of this study is that both the NHSN and physician review were conducted by multiple individuals, which strengthens the internal validity of the study. However, we were limited by relatively small numbers from a single center and the potentially subjective interpretation of cases by physician review, despite being the gold standard. Larger, multicenter studies could confirm our findings and inform future iterations of the NHSN surveillance definitions. Additionally, we would strongly encourage similar validation procedures of future quality metrics such as hospital-onset bacteremia to support their clinical relevance.
